# Editorial: From single stem cells to organoids, organ repair, and public health Volume II

**DOI:** 10.3389/fcell.2023.1294407

**Published:** 2023-09-25

**Authors:** Lon J. Van Winkle, Rebecca J. Ryznar, Philip M. Iannaccone

**Affiliations:** ^1^ Department of Biochemistry, Midwestern University, Downers Grove, IL, United States; ^2^ Department of Medical Humanities, Rocky Vista University, Parker, CO, United States; ^3^ Molecular Biology, Department of Biomedical Sciences, Rocky Vista University, Parker, CO, United States; ^4^ Departments of Pediatrics and Pathology, Lurie Children’s Hospital, Stanley Manne Children’s Research Institute, Northwestern University’s Feinberg School of Medicine, Chicago, IL, United States

**Keywords:** organoid, organ-on-chip, stem cell, organ repair, tissue repair, public health, precision medicine

## Introduction

Soon after pluripotent embryonic stem cells (ESCs) were isolated from preimplantation embryos, the cells were grown individually into 3 dimensional embryoid bodies and, later, organoids, assembloids and “organs-on-chips.” These models recapitulate embryonic and adult tissues in organs *in vivo*. This approach also is used now to produce these mimics of small organs utilizing individual induced pluripotent stem cells (iPSCs). Normal tissue and organ biochemistry and physiology as well as tissue interactions and cross talk are faithfully reproduced in each of these structures. Hence, they have become legitimate models of both physiologic state and development. When iPSC from disease states are used to produce these structures, they can be used to study the details of individuals’ pathologies and possible therapeutics. Papers contributed to this Research Topic describe how stem cells, organoids, assembloids, and organ-on-chip models may soon foster public health through precision medicine and clinical interventions.

## Organoid, assembloid, and organ-on-chip models: future clinical contributions to precision medicine and public health

In their paper for this Research Topic, Goldrich and associates review a process to study the complex interactions among tissues and organs by using ESCs and iPSCs to form three-dimensional tissue-and organ-containing structures *ex vivo* (Goldrick et al.). When the iPSCs are isolated from humans and other mammals with genetic diseases, they also serve as models of these diseases. For example, in Hirschsprung’s disease, a congenital lack of enteric nerve development can lead to an almost complete absence of GI tract motility and insufficient nutrient absorption. And these abnormalities are recapitulated in organ-like structures formed from stem cells with the Hirschsprung’s disease-causing mutations in *PHOX2B* (Workman et al., 2017). One in 5,000 live births are affected by Hirschsprung’s disease (Amiel and Lyonnet, 2001).

Interestingly, such diseases can likely be corrected through transplantation of mutation-free stem cells into affected regions of the GI tract (Pan et al., 2022). Such stem cells can be isolated from donors, or they might be derived from the person with, say, Hirschsprung’s disease using CRISPR/Cas9 gene-editing of their iPSCs. To get enough stem cells to transplant, the cells can be grown *in vitro* in conditions being developed to maximize cell production. In addition, different cell cultures might be pooled to gain the necessary number of cells for transplantation. In this regard, conditions for cryopreservation and storage of viable neural stem cells are being developed as described by Heumuller-Klug and associates in a paper contributed to this Research Topic (Heumüller-Klug et al.). Their results show that cryopreservation of these stem cells is possible although, as of now, with reduced viability. And there was no detected effect of such preservation on the functions of resultant nervous system cell subtypes.

In another effort to form functioning neuronal cells in organoids, Forrester-Gauntlett and associates conclude in their paper for this Research Topic that epithelial integrity is essential to proper formation of inner ear-like organoids (Forrester-Gauntlett et al.). While *Grhl*-knock out in mouse ESCs apparently led to this diminished epithelial integrity, *Grhl*-knock out cells otherwise developed normally expressing neural, neuronal, and sensory hair cell markers. These studies are important because mutations in this transcription factor gene, *GRHL2*, in humans are associated with sensorineural deafness. Moreover, such hereditary hearing loss may soon be treatable through gene therapy including gene replacement, suppression, and editing (Jiang et al., 2023).

Using a simpler approach, Gerwinn and associates formed three-dimensional microtissues from detrusor smooth muscle cells of pediatric patients with end stage lower urinary tract dysfunction. In the process, these smooth muscle cells lost their characteristic disease phenotype, as the authors report in another paper for our Research Topic (Gerwinn et al.). Hence, these three-dimensional structures might be an autologous source of cells for cell-based bladder tissue engineering. Nevertheless, the mechanisms of recovery of apparently normal smooth muscle cell contractility in microtissues remains to be determined. Moreover, such muscle cells seem unlikely to benefit patients with neurogenic bladder dysfunction (Topoliova et al., 2023) as is the case also for smooth muscle cells associated with the neurogenic disorders discussed above.

Finally, *in vitro* derived smooth muscle stem cells were used to partially correct lower esophageal sphincter function in a rat model of gastroesophageal reflux disease—as described in the Research Topic paper by Zhang and associates (Zhang et al.). These smooth muscle stem cells were derived from the rat adipose-derived stem cells these authors used originally to alleviate inflammatory bowel disease (Chen et al., 2013). Interestingly, adipose-derived stem cells may foster nerve regeneration and revascularization of nervous tissue (Saffari et al., 2022), so their inclusion as part of the therapies for neuronal development, described above, might improve some or all of these treatments.

## Conclusion

The juxtaposition of new stem cell biology and 3-dimensional growth technologies has led to a revolution in regenerative biology. Based on the ability to produce stem cells from adult tissues of individuals, the ability to recreate functional tissues has burgeoned into a robust approach to disease physiology, therapeutic test beds, and organ tissue replacement. Importantly, the creation of screening systems with tissue arrays provides direct approaches to minimally invasive autologous organ tissue. Ultimately, combined with gene editing technology direct correction of genetic disease states to homeostatic states will be possible. These exciting advances are explored in the suite of papers presented here and promise a new generation of biologic approaches to disease amelioration.
